# Biological sex identification in the endangered dusky gopher frog (*Lithobates sevosa*): a comparison of body size measurements, secondary sex characteristics, ultrasound imaging, and urinary hormone analysis methods

**DOI:** 10.1186/s12958-016-0174-9

**Published:** 2016-08-02

**Authors:** Katherine M. Graham, Andrew J. Kouba, Cecilia J. Langhorne, Ruth M. Marcec, Scott T. Willard

**Affiliations:** 1 Department of Biochemistry, Molecular Biology, Entomology and Plant Pathology, Mississippi State University, Mississippi State, Starkville, MS 39762 USA; 2 Department of Wildlife, Fisheries and Aquaculture, Mississippi State University, Mississippi State, Starkville, MS 39762 USA

**Keywords:** Sex identification, Body size, Secondary sex characteristics, Ultrasound, Urinary hormone analysis, Dusky gopher frog, Mississippi gopher frog, *Lithobates sevosa*

## Abstract

**Background:**

Accurate sex identification techniques are important for wildlife demographic studies and for genetic management of captive breeding colonies. Various non-invasive methods for identification of biological sex in the weakly dimorphic endangered dusky gopher frog (DGF; *Lithobates sevosa*) were explored to support planned recovery efforts for this species including breeding and augmentation of wild populations.

**Methods:**

Body size (snout-vent length and body weight) measurements, observation of nuptial pads, ultrasound imaging, and urinary hormone analysis for testosterone and estrone were performed on 27 male and 19 female DGFs. For each method, the mean and range of measurement values were determined for male and female DGFs housed in a captive breeding population. The ability of these methods to accurately predict the true biological sex of the individuals was assessed retrospectively.

**Results:**

Body size measurements were of limited use for sex identification purposes, as males and females demonstrated overlapping body lengths and weights. Observation of the presence/absence of nuptial pads in males and females, respectively, proved to be accurate and easy to perform in most cases. Ultrasound imaging was useful for predicting the sex of female frogs, particularly when females were gravid. Commercial enzyme immunoassay kits were validated to measure urinary hormones in the DGF. Mean urinary testosterone (males: 2.22 ± 0.38 ng/ml; females: 0.92 ± 0.11 ng/ml) and estrone (males: 0.08 ± 0.01 ng/ml; females: 1.50 ± 0.39 ng/ml) concentrations were significantly (*p* < 0.05) different between the sexes. However, there was some overlap in hormone concentrations between the sexes. When a ratio of testosterone (T) to estrone (E) concentrations was calculated for each individual, males demonstrated significantly greater T/E ratios compared to females (*p* < 0.05). Use of this ratio showed greater accuracy in predicting the sex of the animal compared to using testosterone or estrone concentrations alone.

**Conclusions:**

Monitoring for presence/absence of nuptial pads and using urinary testosterone to estrone hormone ratios were the most accurate methods for identifying the biological sex of adult DGFs. Urinary hormone measurements for sex identification may be useful in other weakly dimorphic and monomorphic amphibian species in both *ex situ* and *in situ* settings.

## Background

An increase in the number of captive assurance colonies for amphibians has occurred in the last several decades as a conservation measure to combat the drastic global decline and extinction of many species [1–3]. The primary goals for these assurance colonies are to genetically manage the species, reproduce animals for sustainability of the population, and produce offspring for reintroduction programs [1]. Therefore, an obvious but necessary initial management step for meeting reproduction and genetic management goals is to identify the biological sex of the individuals present within an *ex situ* population. However, this is not always easily accomplished, particularly when working with species that have monomorphic or weakly dimorphic secondary sexual characteristics. Without accurate and non-invasive tools to identify the sex of the individuals, researchers may struggle to appropriately pair animals for breeding, risk aggression due to improper housing of groups, or waste time and resources when attempting to collect gametes from inaccurately sexed animals [4, 5].

A captive breeding program for the critically endangered dusky gopher frog (DGF; *Lithobates sevosa*; also referred to as the Mississippi gopher frog) was first established in 2001; however, 15 years later this species has failed to breed naturally in captivity without the intervention of assisted reproductive technologies (ART), such as hormone therapy to stimulate gamete production and *in vitro* fertilization (IVF). Assisted captive breeding efforts were initially hindered by difficulties accurately identifying the biological sex of juvenile and young adult DGFs due to their weak dimorphic characteristics. The DGF is considered weakly dimorphic in captivity, as the adult males often fail to show secondary sex characteristics such as prominent nuptial pads or throat sacs, and the two sexes appear to overlap in size, coloration, and other physical features [5, 6]. In the wild, dark nuptial pads can frequently identify adult males when they are captured migrating to breeding ponds [6, 7]. It is unclear why captive males may fail to display secondary sex characteristics; however, it may be that artificial habitats are missing important environmental cues that could result in lower steroid concentrations leading to poorer representation of these secondary sexual characteristics [5, 7].

Many anurans display some form of sexual dimorphism, including differences in size, skin coloration/texture, secondary sex characteristics (nuptial pads, vocal sac color, spines, glands, etc.), or behaviors [7], allowing researchers to distinguish between males and females. For those anuran species that are weakly dimorphic or monomorphic, a number of strategies for sex identification exist; however, they range in effectiveness and invasiveness. Size dimorphism, including body length and body weight, is a commonly used strategy, with snout-vent length (SVL) or snout-urostyle length (SUL) being two commonly used body length measurements. Mature females tend to be larger in size than males in approximately 90 % of anuran species characterized to date [8, 9]; however in practice, size measurements often fail to be completely discrete between males and females, and thus only a few species consistently demonstrate true size dimorphism [4, 10, 11]. Other physical differences between the sexes, such as the development of secondary sexual characteristics, are frequently seen only on males, with nuptial pads and vocal sacs being two of the most distinguishing characteristics in male anurans [7]. These characteristics are moderated by steroid hormones and may be more or less visible based on the time of year and hormone concentrations relative to breeding season [7]. Behaviors, such as advertisement calling, can also be used for sex identification purposes, but are frequently observable only during the breeding season and are often missing from captive environments [7].

Over the past several years, there has been an increase in the number of studies using non-invasive or minimally-invasive fecal [12, 13] and urine [14–17] steroid hormone analysis for sex identification in anurans. Results varied based on species, season, sample type, and the hormones analyzed, but these studies have shown that hormone analysis may be an accurate method to identify sex in anurans, even with seasonal fluctuations in hormone concentrations. Seasonal sex steroid hormone profiles have also been successfully studied in anurans using blood samples [17–22]. However, anuran blood sampling typically requires invasive sampling procedures, such as cardiac sticks, or sacrifice of the animals, therefore non-invasive endocrine measures should be utilized when possible, particularly for endangered species [14, 23].

Ultrasonography has been used minimally in amphibians, and has been performed primarily for medical diagnostic purposes [24–26]; however, ultrasonography has potential for use in sex identification of anurans, similar to its use in reptiles [27, 28] and fish [29]. Ultrasonography has been used to successfully identify the sex of larger salamanders, including hellbenders (*Cryptobranchus alleganiens*) [30] and Chinese giant salamanders (*Andrias davidianus*) [31]. In smaller anurans, ultrasound has been used minimally to study the reproductive state of females [32], but using ultrasonography for sex identification of anurans has not been common practice [33]. The testes of frogs are small and difficult to visualize via ultrasound, yet developing ovarian follicles may be visible during imaging of a female, particularly if she is gravid [24–26]. In non-gravid females, the reproductive organs can be difficult to discern via ultrasonography [24], and sex identification can remain complicated. Near infrared reflectance (NIR) spectroscopy, a technique which measures the characteristic absorption patterns produced by the vibrations of particular chemical bonds [34], may also show promise as a method to identify sex of anurans, including the DGF [35], although further studies in this field are needed. Both ultrasonography and NIR are non-invasive and require only a brief scan of the animal’s abdomen, but the equipment necessary for these techniques can be relatively expensive and may require a trained technician for analysis. Other techniques, such as endoscopic and laparoscopic evaluation, have been performed in amphibians for sex identification purposes [5, 33, 36], but these techniques are invasive and can be dangerous depending on the species, which limits their use, particularly for endangered species. Lastly, genetic analysis for sex identification purposes has proved to be complicated in amphibians. Despite the fact that amphibians have genetically controlled sex determination, most amphibians do not have distinct sex chromosomes [37, 38], and several oddities such as aneuploidy and polyploidy have been observed [39, 40]. Therefore, genetic analysis is likely to be a difficult and costly method for sex identification.

In order to genetically manage and maintain sustainable *ex situ* populations of the critically endangered DGF, non-invasive strategies for biological sex identification should be evaluated. The objective of this study was to characterize the accuracy and ease-of-use of several minimally and non-invasive sex identification techniques in the DGF, including: measurements of body length, body weight, presence/absence of nuptial pads, ultrasonography, and urinary hormone analysis. These techniques may also be applicable to other weakly dimorphic and monomorphic amphibian species, and may contribute to increased output within captive breeding programs by determining the most effective and accurate sex identification techniques. Researchers utilizing these techniques must find a balance between the need for an accurate answer, and the ease and cost associated with the various methods tested.

## Methods

### Animals and husbandry

A total of 27 male and 19 female DGFs were utilized in this study. Animals were housed at Mississippi State University’s (MSU) Amphibian Conservation Lab (Starkville, MS, USA) for the duration of the study. All animals were captive reared at the Memphis Zoo and Omaha’s Henry Doorly Zoo, and were transferred to MSU prior to the start of the study. Animal ages ranged from approximately 4 to 6 years old throughout the study period. The animal husbandry practices and treatment protocols were approved by Mississippi State University’s Institutional Animal Care and Use Committee (IACUC #10–082).

Frogs were housed in plastic polycarbonate tanks (46 × 66 x 30 cm; Habitat Systems Limited, Des Moines, Iowa, USA) in both single and mixed-sex environments with between one and four conspecifics. Animals were previously implanted with passive integrated transponder tags (PIT tags) allowing for consistent identification of individuals throughout the study. Approximately half of the tank was covered with moistened organic moss, and a small plastic hide (Medium Reptile Hide; LLL Reptile and Supply Co, Oceanside, CA) was provided as additional cover for the frogs. Frogs had access to aged tap water in bowls and were kept on a natural light cycle (Starkville, MS, 33.4625° N, 88.8200° W) throughout the study. Prey items (alternating between mealworms, wax worms, and crickets) were offered three times per week. Mealworms and wax worms were gut loaded with vitamin supplement (Repashy “Superload”; Repashy Ventures Inc., Oceanside, CA) prior to distribution. Worms were offered in small plastic dishes in the tanks. Crickets were gut loaded with fresh fruits and vegetables lightly coated with the vitamin supplement, and crickets were dusted with calcium (Fluker’s Calcium with D3; Flucker Farms, Port Allen, LA) before distribution to the tank.

### Identification of true biological sex

At the start of the study, the biological sex of only a subset of the animals (*n* = 17) was known. Measurements for all methods were collected as if the sex of the animal was unknown or performed blind when possible. Ultimately, the true biological sex of each frog included in the dataset (*n* = 46) was determined based on the production of gametes: where males were observed to produce spermic urine (sperm viewed under 400x magnification on a phase-contrast microscope) and females were observed to produce eggs.

Since the DGFs used in this study were from a captive breeding colony, they were administered exogenous hormone treatments to induce sperm and egg production for use in IVF, and data on the true biological sex of an individual were collected opportunistically as the animals were included in the breeding trials. To induce egg production, females were administered an exogenous hormone treatment once per month for up to three months, until eggs were expressed. This treatment regime was performed once in the late summer or fall (August, September, October, November) and again in the spring (February, March, April). Males were administered an exogenous hormone treatment up to once per month to induce spermiation. Female and male gopher frogs were administered a gonadotropin releasing hormone agonist (GnRHa; #L4513, Sigma Aldrich, St. Louis, MO), or GnRHa in combination with human chorionic gonadotropin (hCG; #C1063, Sigma Aldrich, St. Louis, MO), in order to procure gametes. The treatments followed those reviewed by Kouba et al. [5, 41] for successful egg and sperm production in various *bufonids* and *Rana pipiens*. Our research group has found that female DGFs rarely spontaneously deposit eggs following hormone treatments; therefore, eggs were manually expressed from the females using gentle stimulation 2–3 days post hormone administration. Males produced spermic urine between 0.5 and 2 h post-hormone treatment with a 100 % response rate. Two females and two males died during the study period (from non-experiment related causes); therefore, these individuals only had weight and nuptial pad data through November 2014, and did not have sufficient urine samples to include in the hormone analysis. These animals were necropsied following death, and the sex of the two females was confirmed based on the presence of ovaries and developing oocytes (the males had already produced gametes by the time of death, whereas the females had not).

In this study, data for the subsequent sex identification techniques (body size, secondary sex characteristics, ultrasonography, and urine samples for hormone analysis) were collected prior to the start of the exogenous hormone treatments, or when possible, prior to the administration of hormone treatments in the fall and spring months. Detailed information about the timing of data collection relative to the hormone treatments can be found in the subsequent methods sections. The accuracy of each sex identification method was assessed by calculating the percent of animals whose sex was correctly classified by the method when compared to their true biological sex as confirmed by gamete production.

### Body size measurements

Body length was assessed using snout-vent length (SVL), which measures the distance from the tip of the snout to the vent (cloaca) of the frog. Frogs were placed on a small plastic dish and SVL was measured to the 0.1 millimeter (mm) using dial calipers (#134160001; Bel-Art Products, Wayne, NJ). All measurements were taken by a single observer to avoid inter-observer variability. The SVL measurements were conducted at the start of the summer study period (July, 2014) and in spring (Feb, 2015), prior to exogenous hormone administration. There was no significant difference in the means of the SVL measurements, and there was no consistent trend of an increase or decrease in SVL within individuals over the study period, therefore the SVL measurements were averaged for each animal. The mean standard error (SE) between SVL measures within an individual was 0.9 mm. Body weight (BW) was measured approximately once per week to the 0.1 gram (g) during the study period (July 2014-June 2015) as part of the weekly animal care routine. Weight measurements were averaged for each individual during the study period.

Mean (±SE) SVL and BW for each sex was calculated at the end of the study period based on the confirmed sex of the animals. Following verification of data normalcy (Shapiro-Wilk test), a two-sample *t*-test was used to test for differences between the sexes (male and female) for average SVL and BW. Significance was set at *p* < 0.05, and the unpooled variances (Satterthwaite) test statistic was used based on a significant Fold-F value in the Equality of Variances test. Statistical analysis was performed using SAS 9.4 (Cary, NC). The accuracy of using body size measures to predict the sex of DGFs was then assessed. For SVL, accuracy was determined by calculating the overall mean of SVL measurements for both sexes, and this mean value was used as a boundary for predicting sex, such that any animal exhibiting an SVL measurement above the mean was classified as “female” (as the mean female SVL was greater than the mean SVL for males), and any animal with an SVL measurement below the overall mean would be classified as “male”. The percentage of animals whose sex was correctly predicted using this SVL boundary was calculated. The same calculation was performed to determine the accuracy of using body weight for sex identification, where the overall mean of body weight measurements for both sexes was determined, and this mean was used as the boundary for predicting sex. Any animal with a body weight measurement above the mean was classified as “female” (as the mean female body weight was greater than the mean body weight for males), and any animal with a body weight measurement below the overall mean was classified as “male”. The percentage of animals whose sex was correctly predicted using this body weight boundary was calculated.

### Secondary sexual characteristics

A common secondary sex characteristic of male anurans are raised, keratinized patches of skin on the fingers called nuptial pads, or thumb pads [7, 38]. As these pads are under hormonal control and can vary by season [42], a check for nuptial pads was made on each individual once per month for 12 months by examining both thumbs for a darkened, raised patch of skin. A single observer collected data, and the presence or absence of pads for each month was recorded. Observations for nuptial pads occurred prior to any hormone administration for the month when possible, however, in some cases, a male was administered hormones prior to the monthly nuptial pad check because sperm was needed to fertilize eggs for IVF. Based on nuptial pad data, an animal was classified as “male” if it exhibited nuptial pads for greater than 50 % of the observations. The percentage of animals whose sex was correctly predicted based on presence/absence of nuptial pads was calculated overall and for each sex.

### Ultrasonography

A trans-abdominal ultrasound scan of each individual was performed at the start of the study (July 2014) for sex identification purposes. This measure was performed once, prior to any exogenous hormone treatments, as the hormone treatments and subsequent egg deposition would have artificially altered the appearance of the ovaries and follicles in the female. Imaging was performed with a Sonosite MicroMaxx ultrasound (Sonosite Inc., Bothell, WA) equipped with a 38-mm broadband linear array transducer (range 6–13 MHz) set to a scan depth of 2.7 cm on the breast/tissue setting. The abdomen of the frog was moistened with aged tap water prior to ultrasonography for use as a conductor to enhance imaging. During the scan, an assessment of sex was made based on the visualization of any reproductive organs (testes or ovaries) or the presence of developing follicles on the ovary. A single observer who was experienced at ultrasound imaging of amphibians performed ultrasonography and assessment of images. The observer was blind to the true sex of the animals to avoid bias. The percentage of animals whose sex was correctly identified based on ultrasound imaging was calculated overall and for each sex.

### Urinary hormone analysis

Urine samples were collected from DGFs (*n* = 25 males; *n* = 17 females) to test for differences in hormone concentrations based on biological sex. Samples were collected during two different months (February and July) to account for potential seasonal differences in hormone concentrations between breeding season (February) and non-breeding season (July) [6]. Urine samples were collected prior to any exogenous hormone treatments given during these months. Urine was collected by holding the frogs over a large plastic dish and gently inserting a small piece of vinyl catheter tubing (#BB31785-V/5; Scientific Commodities Inc., Lake Havasu City, AZ) into the cloaca to draw urine into the dish. Upon urination, the sample was pipetted into a 1.5 ml centrifuge tube (#05-408-129; Fisher Scientific, Pittsburgh, PA) before being stored at -20 °C prior to hormone analysis.

Two hormones, testosterone and estrone, were selected for potential sex identification purposes based on their validation in several other anuran species [14–16], and based on preliminary hormone analysis work with boreal toads (*Anaxyrus boreas boreas*) in our lab (Graham, unpublished data). Immunoreactive testosterone, estrone, and their metabolites (henceforth referred to simply as testosterone and estrone) were measured using commercially available enzyme immunoassay (EIA) kits. Kits used for analysis included DetectX Testosterone Enzyme Immunoassay kits (#K032; Arbor Assays, Ann Arbor, MI) and DetectX Estrone Enzyme Immunoassay kits (#K031; Arbor Assays, Ann Arbor, MI). Manufacturing information for the assay kits stated the cross reactivity for the testosterone antibody was 100 % with testosterone, 56.8 % with 5α-dihydrotestosterone, 0.27 % with androstenedione, and less than 0.05 % with androsterone, DHEA, cholesterol, estradiol, progesterone, pregnenolone, hydrocortisone, cholic acid, and cholic derivatives; while the estrone antibody had a cross reactivity of 100 % with estrone, 112 % with estrone-3-glucuronide, 65.5 % with estrone 3-sulfate, 5 % with estradiol, and less than 0.1 % with estradiol-3-sulfate, estriol, progesterone, pregnandiol, cortisol, and androsterone. The manufacturer stated assay sensitivities to be 9.92 pg/ml for the testosterone kit and 22.4 pg/ml for the estrone kit. Optical densities of the wells were read using a SpectraMax Plus 384 microplate reader (Molecular Devices, Sunnyvale, CA) at a 450 nm wavelength. Assay data and validation tests were analyzed using the free online analysis program MyAssays (MyAssays Ltd., Brighton, Sussex, UK), using the Arbor Assays testosterone and estrone EIA data templates or with GraphPad Prism 6 (Version 6.03, GraphPad Software, Inc., La Jolla, CA).

As DGF urine had not been analyzed on these EIA systems previously, several validation tests were performed prior to hormone analysis of individual samples. Separate pools of confirmed male and female urine samples were serially diluted and compared to the standard curve to test parallelism for each assay. An F-test was used to confirm parallelism, where a non-significant (*p* > 0.05) value demonstrated the slopes of the curves were parallel. A successful parallelism test indicates the assay can bind the hormone of interest across multiple dilutions in a predictable manner [43]. In addition, an accuracy test (in which a pool of low concentration samples was spiked with known standard hormone concentrations) was performed for each assay system to confirm the urine matrix did not cause interference in the assay system [43]. The recovery of hormone was expressed using the linear regression formula of y = mx + b, where a slope (m) greater than or less than 1, represents an over or underestimation of the hormone of interest, and a range of slopes between 0.8 and 1.2 is generally considered to be acceptable accuracy [23, 43].

Individual sample values for each hormone were reported in ng/ml, and were adjusted for specific gravity as measured by a digital urine specific gravity refractometer (#PAL-10S; Atago USA Inc., Bellevue, WA), as specific gravity can be used in place of creatinine measures to account for variation in hydration [44, 45]. Prior to statistical analysis, hormone values (adjusted for specific gravity) were log transformed. A multivariate repeated measures analysis was used to test for significant differences between the month of collection (February and July) and sex (male and female). Because no significant difference was found based on month of collection (*p* > 0.05), the values for February and July were averaged for each hormone to produce a single mean testosterone and estrone value for each animal. Two-sample t-tests were then used to test for differences in testosterone and estrone concentrations between males and females. If the equality of variances test was violated (as indicated by a Folded F statistic of *p* < 0.05), the Satterthwaite test statistic was used to adjust for unequal variances. The accuracy of using testosterone and estrone concentrations for sex identification was assessed similar to the body size measures, where the overall mean for each hormone was calculated and used as a boundary for sex prediction. Any animals exhibiting testosterone concentrations above the overall mean value were classified as “male”, while any animals exhibiting estrone concentrations above the overall mean value were classified as “female” based on the fact that the mean testosterone concentration was greater in males and mean estrone concentration was greater in females.

In addition to comparing the mean testosterone and estrogen concentrations between males and females for sex identification, a unit-less ratio of testosterone to estrone (T/E) was also calculated for each animal, similar to previous studies [12, 13, 46]. A multivariate repeated measures analysis was again used to test for differences based on the month of collection (February and July) and sex (male and female) for the T/E ratios. Because month of collection was not significant, the final T/E ratio used for analysis was calculated using the averaged testosterone and estrone values described above. The accuracy of using the T/E ratio for sex identification was assessed using the methods previously described, where the overall mean T/E ratio for both sexes was calculated, and this value was used as a boundary for predicting sex. Since males demonstrated higher mean testosterone and lower mean estrone compared to females, it would be expected that males should have a greater T/E ratio, and thus, any animal with a T/E ratio greater than the overall mean T/E ratio for both sexes was classified as male, and any animal with a T/E ratio less than the overall mean T/E ratio for both sexes was classified as female. The percentage of animals whose sex was correctly predicted using this T/E ratio boundary was calculated.

## Results

### Body size measurements

**Fig. 1:**
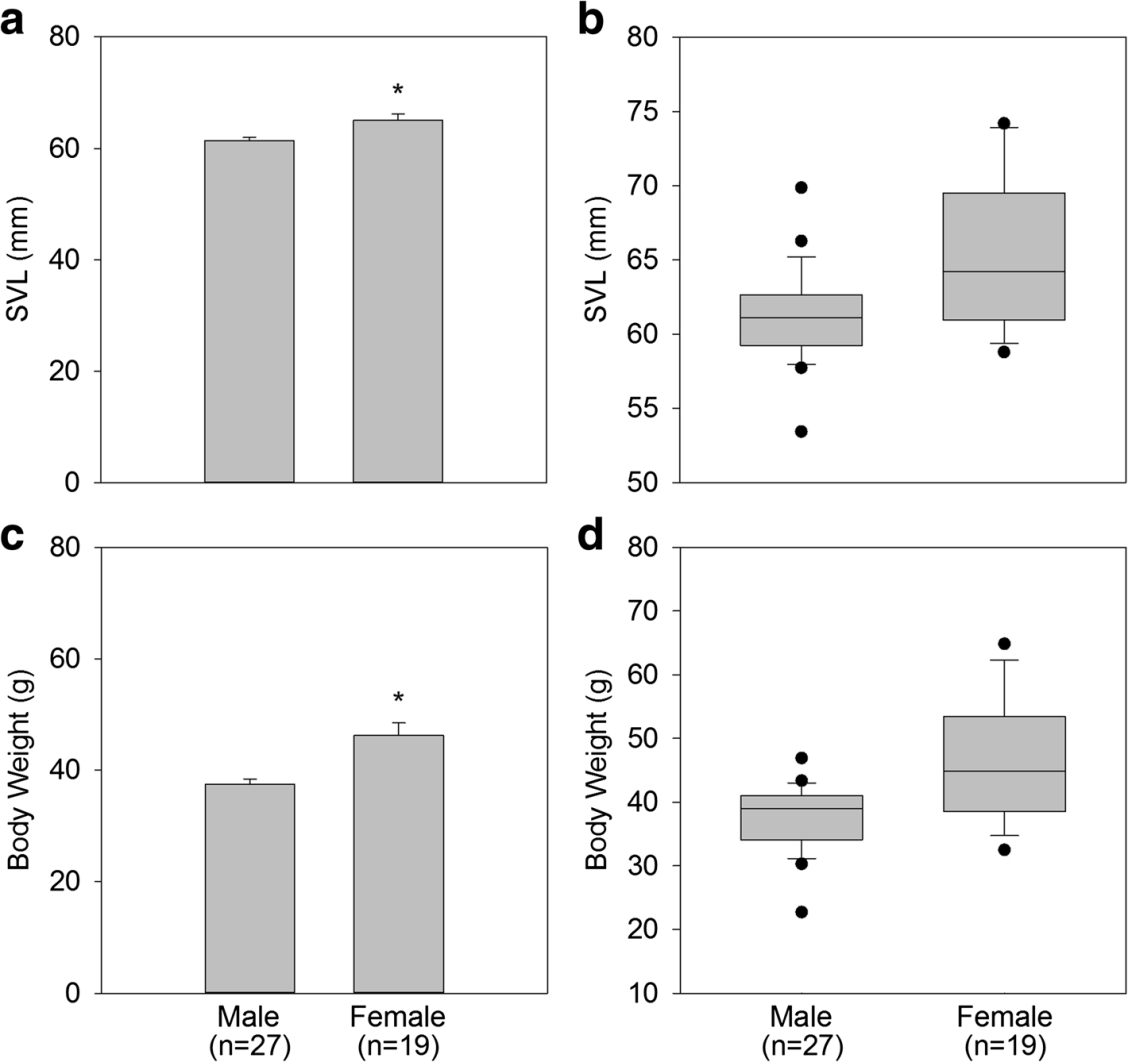
Comparisons of snout-vent-length (SVL; mm) and body weight (g) measurements for male and female DGFs. Mean SVL (Panel **a**) and body weight (Panel **c**) were significantly different (*, *p* < 0.05) between males and females, with females demonstrating greater mean SVL and body weight. Boxplots for SVL (Panel **b**) and body weight (Panel **d**) demonstrate there was a large overlap between males and females for both measures. In panels **b** and **d**, boxes show the quartile 1 to quartile 3 ranges with the horizontal line indicating the median. Whiskers show the 90^th^ and 10^th^ percentile, and the dark circles indicate outliers beyond this range

Mean SVL was 61.3 ± 0.6 mm for males (*n* = 27) and 65.1 ± 1.1 mm for females (*n* = 19). There was a significant difference between the sexes for mean SVL (*p* < 0.05; Fig. 1, Panel a). However, there was clear overlap in SVL measures between the sexes, with mean SVL measurements ranging from 53.4 mm to 69.9 mm in males and 58.8 mm to 74.2 mm in females (Fig. 1, Panel b). The mean SVL length for both sexes was calculated to be 62.8 mm. Using this value as a boundary to predict sex (where any frog below 62.8 mm was classified as male and any frog above this threshold was classified as female), SVL was accurate in predicting sex 73.9 % of the time. Specifically for each sex, it correctly identified males 81.5 % of the time and females 63.2 % of the time (Table 1).

**Table 1:** Comparison of accuracy for each tested sex identification method

Method	Overall accuracy	% accuracy: males	% accuracy: females
Body length (SVL)	73.9 %	81.5 %	63.2 %
Body weight	71.7 %	77.8 %	63.2 %
Nuptial pads	95.7 %	92.6 %	100.0 %
Ultrasound	93.4 %	88.9 %	100.0 %
Urinary testosterone	66.7 %	44.0 %	100.0 %
Urinary estrone	83.3 %	100.0 %	58.8 %
Urinary hormones (T/E ratio)	95.2 %	92.0 %	100.0 %

Mean body weight was 37.4 ± 1.0 g for males (*n* = 27), and 46.3 ± 2.2 g for females (*n* = 19). There was a significant difference between the sexes for mean BW (*p* < 0.05; Fig. 1, Panel c), however, average BW ranged from 22.7 g to 46.9 g in males and 32.5 g to 64.9 g in females, again demonstrating overlap between the sexes (Fig. 1, Panel d). The mean BW for both sexes was calculated to be 41.1 g. Using this value as boundary to predict sex (where any frog below 41.1 g was classified as male, and any frog above this threshold was classified as female), BW was accurate in predicting sex 71.7 % of the time. Specifically for each sex, it correctly identified males 77.8 % of the time and females 63.2 % of the time (Table 1).

### Secondary sexual characteristics

**Fig. 2:**
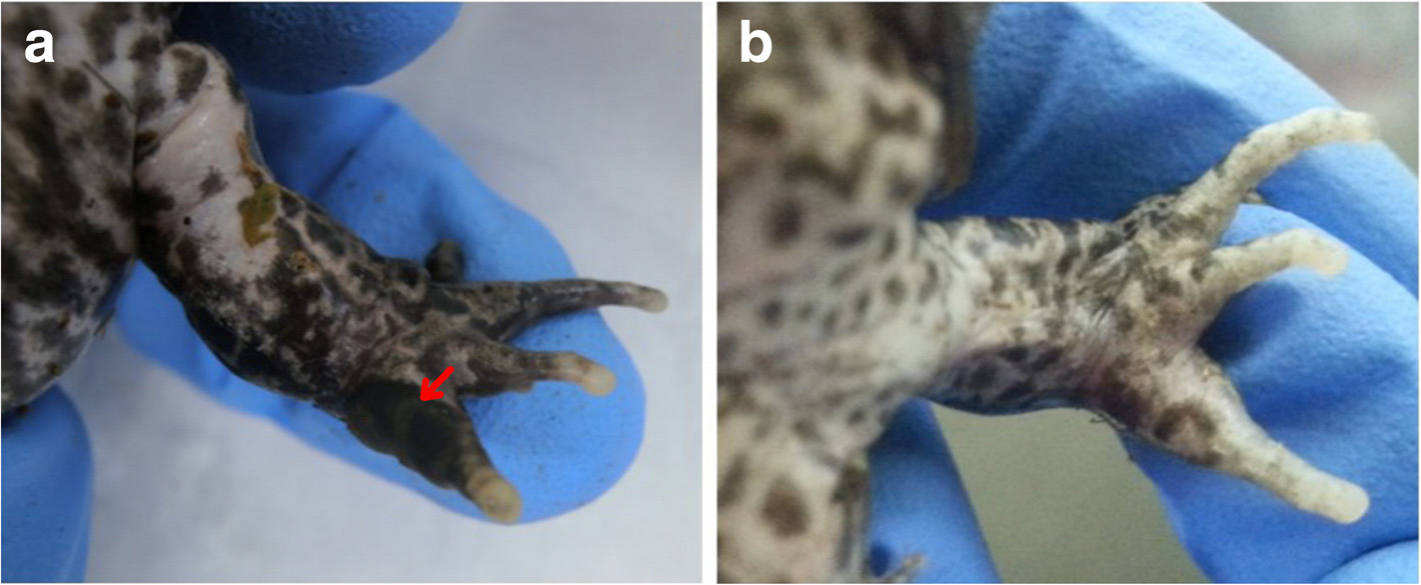
Example of presence versus absence of nuptial pads on DGFs. Panel **a**: male with darkened patch on front digit typical of nuptial pad, as indicated by arrow. Panel **b**: female with no nuptial pad/dark patch on front digit. However, as can be seen in Panel **b**, females have spot patterns on their digits, which can, in some cases, complicate nuptial pad identification

Using the method outlined previously, where an animal was classified as “male” if it exhibited nuptial pads for greater than 50 % of the monthly observations, a total of 44 of the 46 frogs (95.7 %) were accurately sexed. The two cases of misidentification were males, which showed nuptial pads for less than 50 % of the monthly observations. Both of these individuals showed faint presence of nuptial pads at least once during the yearlong study. For one of these misidentified males, the nuptial pads were present for two months in the fall, and for the other male the nuptial pads were present for 2 months in the late winter/early spring. Seven other individuals (both males and females) also showed a change in the apparent presence of nuptial pads during the study, but this was not consistently related to season or length of time since hormone injection. If the seven individuals showing discrepancies in the presence of nuptial pads across the months are considered incorrect (as a conservative measure of accuracy), the percent of correctly identified individuals using nuptial pad data falls to 84.8 %. An example of nuptial pads on a male and absence of pads on a female can be seen in Fig. 2.

### Ultrasonography

**Fig. 3:**
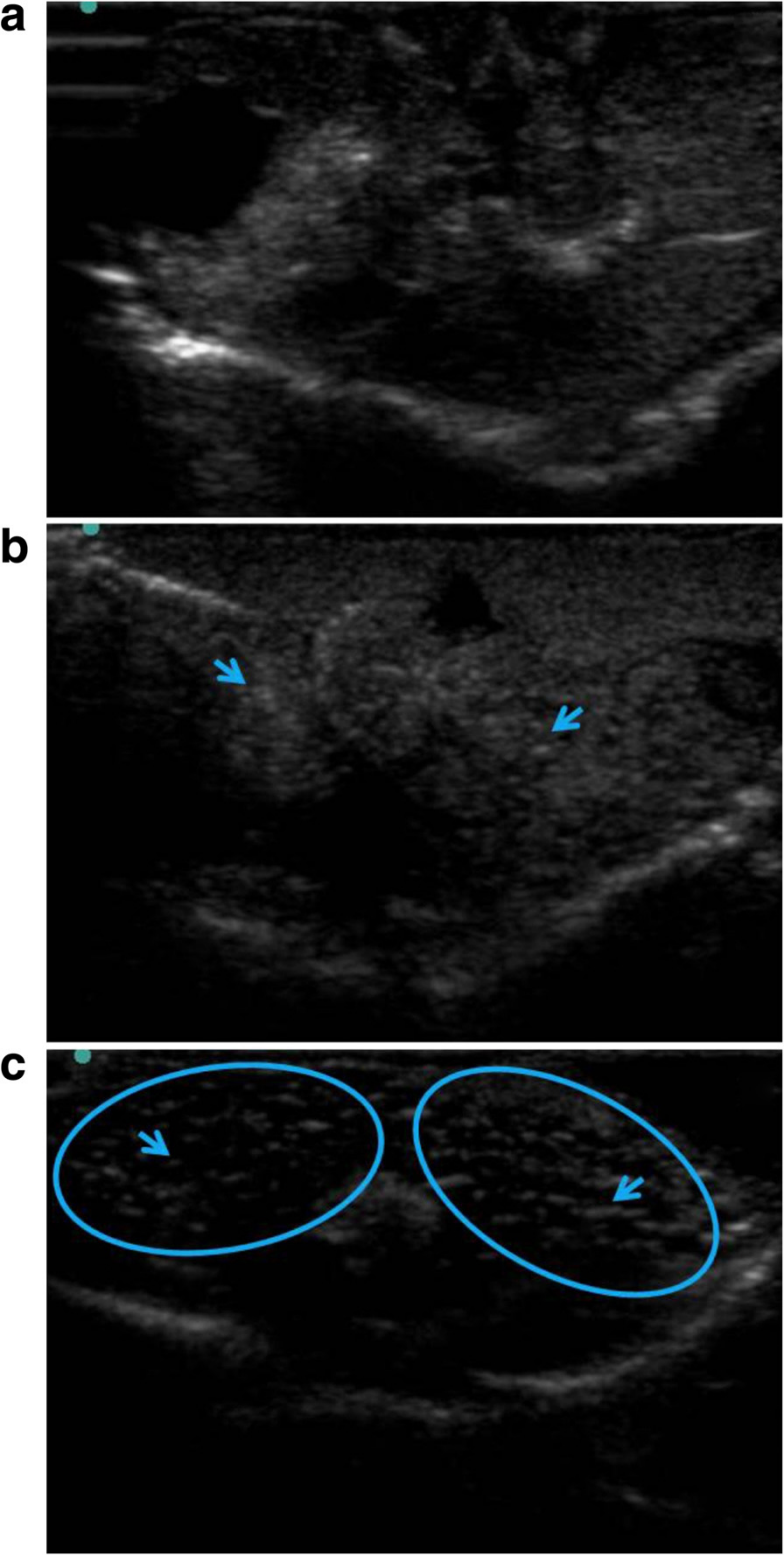
Ultrasonography images used for DGF sex identification. Panel **a** depicts a trans-abdominal ultrasound scan of a male, while Panels **b** and **c** show female scans with low and high follicular development, respectively. Arrows indicate examples of follicles. Large patches of well-developed follicles are circled (panel **c**) to highlight the characteristic spacing and pattern of light and dark areas found in the abdomen of a gravid female. Females with low follicular development generally have smaller follicles and show less space between them (panel **b**)

As expected, testes were difficult to observe in males; however, females with follicles developing on the ovary were relatively easy to recognize based on a characteristic pattern of hyperechoic and hypoechoic (light and dark) areas throughout the abdomen during imaging (Fig. 3). Overall, ultrasound imaging was 93.4 % accurate (43 of 46) in correctly identifying the biological sex of the frogs. Males were correctly identified by ultrasonography 88.9 % of the time, with three instances where a male was classified as a female with low follicular development. Females were correctly identified 100.0 % of the time, with no females being misidentified as males (Table 1).

### Urinary hormone analysis

Validation tests indicated that EIAs reliably measured both testosterone and estrone concentrations in DGF urine samples. Serially diluted pooled samples demonstrated slopes parallel to the standard curve in both assay systems, as indicated by non-significant (*p* > 0.05) F-test statistics (Table 2). Accuracy tests demonstrated significant recovery (*p* < 0.05) of both testosterone (y = 1.0213x-51.9754, r^2^ = 0.9982, *n* = 6) and estrone (y = 0.9913x-22.8574, r^2^ = 0.9957, *n* = 7).

**Table 2:** Results of parallelism validation tests for testosterone and estrone enzyme immunoassays

	Testosterone parallelism		Estrone parallelism	
	*F-test*_*(DFn,DFd)*_	*P-value*	*F-test*_*(DFn,DFd)*_	*P-value*
Male urine pool	F_(1,8)_ = 2.9675	0.12	F_(1,7)_ = 3.1545	0.12
Female urine pool	F_(1,8)_ = 4.0431	0.08	F_(1,10)_ = 1.1846	0.30

*DFn,DFd*: degrees of freedom numerator (DF*n*), degrees of freedom denominator (DF*d*)

**Fig. 4:**
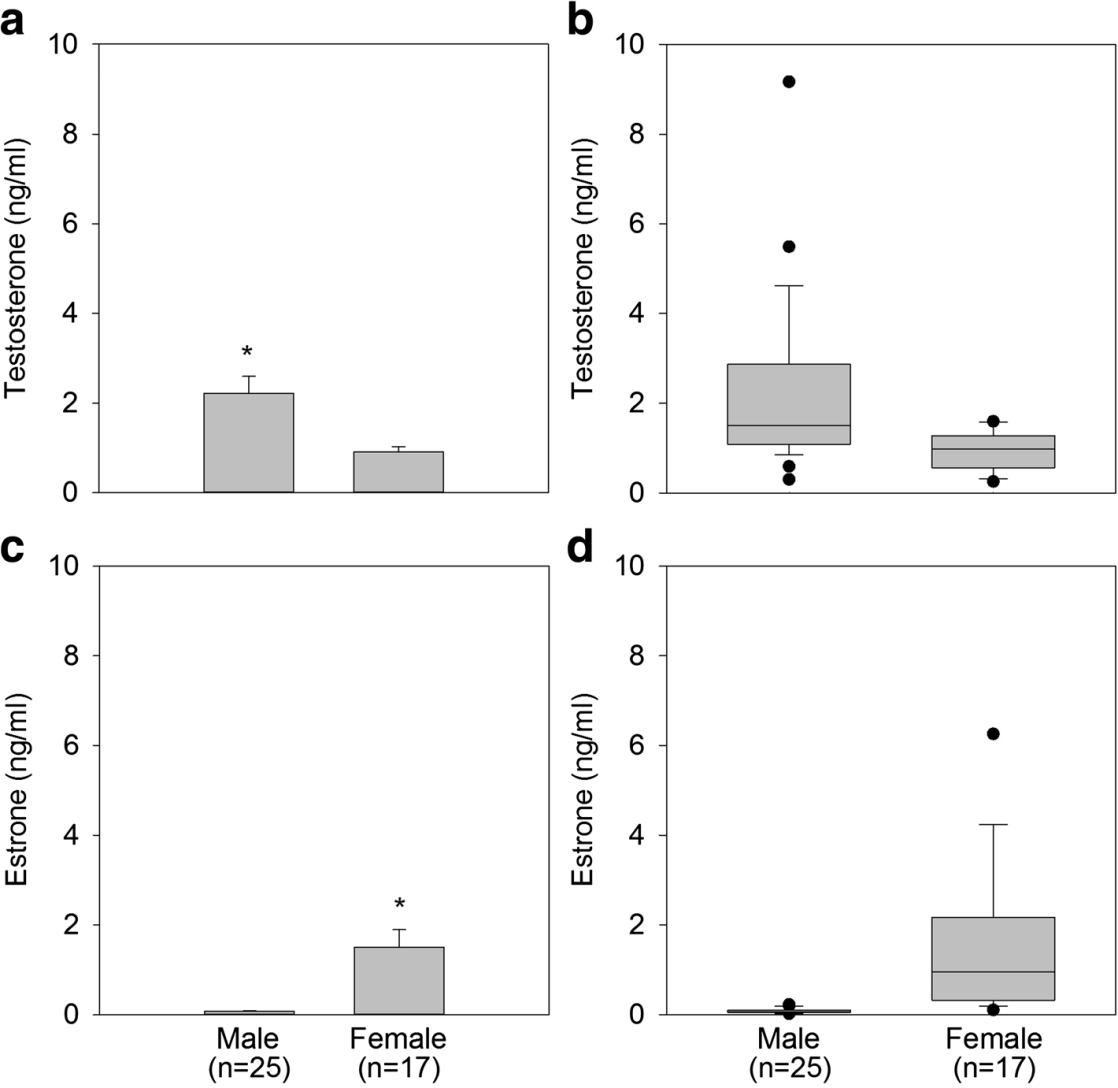
Comparison of mean testosterone (panel **a**) and mean estrone (panel **c**) concentrations for male and female DGFs. There was a significant difference in the mean concentration of testosterone (*, *p* < 0.05) and estrone (*, *p* < 0.05) between the sexes. Boxplots for each hormone are in panels **b** (testosterone) and **d** (estrone), with shaded regions depicting the quartile 1 to quartile 3 ranges, while whiskers indicate the 90th and 10th percentile. Dark circles indicate outliers beyond this range. The horizontal line within the box represents the median

Within each sex (male or female) there was no significant difference in testosterone (*p* > 0.05) or estrone (*p* > 0.05) concentrations between the February (breeding season) and July (non-breeding season) samples. Therefore, the testosterone and estrone concentrations for February and July were averaged to calculate a single testosterone and estrone concentration for each animal. Mean testosterone concentration was 2.22 ± 0.38 ng/ml for male DGFs and 0.92 ± 0.11 ng/ml for female DGFs (Fig. 4, panel a). Testosterone concentrations ranged from 0.30 to 9.16 ng/ml in males and 0.26 to 1.60 ng/ml in females (Fig. 4, panel b). Mean testosterone concentrations were significantly greater (*p* < 0.05) in males compared to females. Mean estrone concentration was 0.08 ± 0.01 ng/ml in males and 1.50 ± 0.39 ng/ml in females (Fig. 4, panel c), with females showing significantly greater (*p* < 0.05) estrone concentrations compared to males. Estrone concentrations ranged from 0.02 to 0.23 ng/ml in males and 0.11 to 6.25 ng/ml in females (Fig. 4, panel d). Although the mean testosterone and estrone concentrations were significantly different between the sexes, there was some overlap in the hormone concentrations of males and females (Fig. 4, panels b and d), particularly for testosterone. The overall mean testosterone concentration for both sexes was 1.69 ng/ml. When this value was used to predict sex, 100 % of females were correctly predicted, but only 44 % of males. For estrone, the overall mean concentration for both sexes was 0.66 ng/ml. Using this value as a boundary to predict sex, males were correctly identified 100 % of the time, but females were only correctly identified 59 % of the time.

**Fig. 5:**
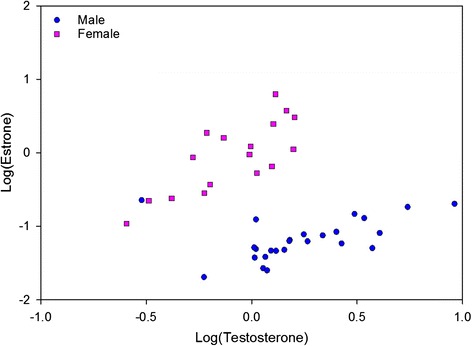
Scatterplot showing the sex of the DGFs can be distinguished by the ratio of urinary testosterone to estrone. Data shown are log transformed. Blue circles indicate males, while females are indicated by pink squares

When using a ratio of testosterone to estrone (T/E) concentrations (concentration of urinary testosterone (ng/ml) divided by concentration of urinary estrone (ng/ml) for each animal), the ability to discriminate between the sexes was improved. Males demonstrated significantly greater T/E ratio values compared to females (*p* < 0.05), with ratio means of 30.5 ± 2.88 for males and 1.15 ± 0.18 for females. As shown in Fig. 5, there is a clear distinction when the testosterone to estrone concentrations are plotted, with the exception of one clear outlier male. The mean T/E ratio for both sexes was 18.63. When this value was used as a boundary to predict sex (where any animal with a T/E ratio greater than 18.63 was classified as male, and any animal with a T/E ratio below this threshold was classified as female), urinary hormone analysis was accurate in predicting sex 95.2 % of the time. Specifically for each sex, it correctly identified males 92.0 % of the time and females 100.0 % of the time (Table 1).

## Discussion

Accurate sex identification of weakly dimorphic and monomorphic anurans is important for genetic management and sustainability of captive breeding programs, as well as for researchers performing *in situ* population assessments. This study compared several different techniques for ease-of-use and accuracy in identifying the biological sex of the critically endangered DGF. Size measurements and assessment of nuptial pads were easiest to perform, although variability in these measures may limit their accuracy. On average, females were larger than males in both SVL and body weight; however, using SVL and body weight measurements to predict the biological sex was only mildly successful due to the wide overlap in measurements between the sexes. In particular, females showed greater variability in SVL and weight, as evidenced by the wide range of values observed. Therefore, using body size measures to predict sex may be most successful when the individual in question has measurements that are at the more extreme ends (low for males and high for females) of the population’s body size range.

The ranges presented in this study are also specific to the DGF population housed at MSU, and these averages may not be applicable to other populations of DGFs, either *ex situ* or *in situ*. For example, size measurements of DGFs at other zoological institutions we have worked with have been larger than the DGFs housed at MSU. At these zoos, males (*n* = 5) have ranged in SVL from 60.8 mm to 71.9 mm, with an average of 64.2 mm, while female (*n* = 8) SVL ranged from 66.8 mm to 79.3 mm with an average of 71.5 mm. Similarly, weight data from males at other zoos ranged from 39.6 g to 64.7 g, with an average of 48.0 g, while females ranged from 49.8 g to 80.2 g, with an average weight of 63.0 g. This variability in reported measurements highlights that making predictions about sex based on body size may be difficult across captive populations at different institutions. Published size ranges for wild DGFs are also variable, with adult males reported to be from 51 to 85 mm and females reported to be 64–94 mm [47, 48]. Comparing body size measurements between *in situ* and *ex situ* populations is further complicated by the fact that animals housed in a captive setting are fed a constant diet, whereas animals *in situ* may have more variable weights due to availability of prey. Moreover, time of year may also play a role in body size measurements, as females of many species can exhibit dramatic weight increases when gravid [49]. For some anuran species, growth is reported to continue throughout adulthood; therefore, the age class of a frog must also be considered when making size measurements [10]. In this study, all the animals were close in age (4–6 years old), so this should not account for differences in the measurements. In addition to variability from environmental variables, SVL has often been criticized as a measure for sex identification because of its high inter and even intra-observer variability [50–52]. The difficulty obtaining a consistent SVL measurement may partially explain the wide range of sizes reported in the literature. In this study, a single researcher performed all SVL measurements to reduce the effects of inter-observer variability.

Predicting sex based on the presence of nuptial pads was generally accurate and easy to perform. The two males misidentified using this method showed nuptial pads in only two of the 12 months; and were therefore classified as females using criteria that an animal needed to display nuptial pads greater than 50 % of the study observations. Interestingly, there were some females that appeared to have nuptial pads during a few months of the study (though not more than 50 % of the time). Nuptial pads would be expected to occur only on sexually mature males; however, because of the dark spotted pattern on the digits of many DGFs there may have been instances where the dark spots on a female’s digits appeared to look like nuptial pads.

Using nuptial pads for sex identification was slightly complicated by the fact that some frogs, both males and females, showed variation in the apparent presence or absence of nuptial pads throughout the year. Many secondary sex characteristics are under the control of steroid hormones in anurans; therefore, they may be more or less visible at certain times of the year [7]. Additionally, the animals were treated periodically with exogenous hormone treatments for separate breeding studies, so this may have affected the visibility of the nuptial pads. Potential changes in the appearance of nuptial pads in relation to the hormone treatments was considered, but appeared to be variable—for example, a male who previously did not have observable nuptial pads in July, August, and September presented with very faint nuptial pads in October within a week of a hormone treatment, and continued to have faint nuptial pads through the following month (29 days post hormone treatment). However, later in the study period (February), the same male received another hormone treatment and did not present with nuptial pads at 8 days post hormone treatment, demonstrating that the hormone treatments did not always coincide with a change in nuptial pad appearance. In another instance, a different male who had previously presented with nuptial pads July-September (and had never been administered hormones during or prior to this time) was not observed to have nuptial pads during the October check, which was performed 9 days after this male was administered hormones. These examples suggest changes in nuptial pad appearance and may not be directly related to exogenous hormone administration. There was also no consistent link between season and any changes in the apparent presence/absence of nuptial pads.

Despite these discrepancies, using the presence or absence of nuptial pads to predict sex was an easy and relatively reliable technique, and is therefore recommended for use as a suitable method for predicting the sex of adult DGFs, or can be utilized if other more advanced sex identification methods are not available. It has been reported that some populations of captive anurans tend to have reduced or absent secondary sex characteristics [5]; however, this did not appear to be a problem for most male frogs in our population. Although, if many of the animals in a captive population appear to lack nuptial pads, the possibility of reduced secondary sex characteristics from a lack of appropriate environmental stimuli should be considered and alternative sex identification methods will need to be employed. Since nuptial pad presence is under hormonal control, reassessing animals periodically for nuptial pads may be worthwhile, particularly during breeding season, or following any hormone treatments, which may cause nuptial pads to appear more distinct [5]. However, our study did not find the season or hormone treatments to be strongly linked to nuptial pad presence for this DGF population, and most animals (39 of 46) did not show a change in nuptial pad presence/absence throughout the study.

Ultrasound technology is likely available at many zoological institutions and universities where frogs may be housed, but it can be extremely difficult to visualize the actual reproductive organs (testes and ovaries) of anurans [24, 26]. While this study found that ultrasound imaging was fairly successful in correctly identifying the sex of DGFs when performed by an observer with extensive amphibian imaging experience, it could be argued that this technique may be of limited use to those researchers with minimal amphibian ultrasound experience. The developing follicles of females are the most visible and easy to distinguish on ultrasound images; therefore, for the average observer, the greatest accuracy in using ultrasound imaging may be limited to females with well-developed follicles. For some anuran species, particularly those that are highly seasonal in their reproduction, successful imaging of the follicles may be limited to certain seasons, as the follicles only grow and develop during particular times of the year [7]. Seasonality did not appear be an issue for DGFs in this study, as females were observed to have follicular development throughout the year based on regular ultrasound imaging (Graham, unpublished data); however, these females were also periodically treated with exogenous hormones for separate studies so it is unknown how this may have affected their normal pattern of follicular growth and development. For this study, only the single ultrasound image from the start of the study (prior to any hormone administration) was used to avoid changes in the ultrasound images of the females associated with the hormonally induced development and deposition of eggs.

This is the first study to provide validation of urinary testosterone and estrone enzyme immunoassays for the DGF. Parallelism and accuracy tests indicated the commercially available assay systems tested here could be used to measure testosterone and estrone (and their metabolites) in DGF urine samples. It is worth noting that the estrone antibody in the Arbor Assays kit had particularly high cross-reactivity with the metabolites estrone-3-sulfate and estrone-3-gluconeride. It is not known if the estrone measurements recorded here were due to high concentrations of the parent hormone estrone, or more likely, its metabolites in DGF urine.

Mean testosterone and estrone concentrations were significantly different between the sexes. However, testosterone concentrations were found to overlap between males and females, and were of minimal use for sex identification purposes. There was also some overlap in estrone concentrations between the sexes, although urinary estrone demonstrated greater clarification between the sexes than did testosterone. This is similar to other anuran studies where estrone glucuronide was found to be higher in females and correctly predicted sex in most cases, while testosterone concentrations showed greater overlap between the sexes [14, 15]. This study found that using a ratio of testosterone to estrone concentrations provided the greatest clarity for sex identification in the DGF. This has also been observed in other anuran studies utilizing hormones for sex identification purposes [12, 13].

It has been found that there are shifts in steroid hormone profiles of some anurans throughout the year based on the state of follicular development, with testosterone and estrogen increased during mid-folliculogenesis and vitellogenesis [53]. Although this study found no significant difference between the two seasons of collection (February, breeding season versus July, non-breeding season), this pattern is worth further exploration especially since seasonal changes in urinary hormones have been observed in other anuran species [14]. Patterns of hormonal seasonality for the DGF are difficult to assess based on the results of this study because the DGFs had received exogenous hormone therapy for reproductive purposes, and it is not known how these treatments may have affected seasonal profiles. However, the DGFs had not been treated with hormones for several weeks to months prior to urine collections for this study; therefore, it is unlikely that endogenous estrone and testosterone concentrations measured in the urine samples were artificially elevated in response to exogenous hormone therapy. Future studies to monitor urinary hormones in wild populations of DGFs may help elucidate possible seasonal hormone patterns for this species.

This study measured only two hormones for sex identification purposes. Future studies should consider testing other hormones such as estradiol, progesterone, or dihydrotestosterone for sex identification, although using a ratio of testosterone to estrone appears to accurately identify the sex of DGFs in most cases. A disadvantage to hormone analysis is the requirement of a laboratory and equipment necessary to analyze assays, and that each new species, sample type, and assay system must have the proper validation tests in order confirm the assay is accurately measuring the hormones of interest [17, 23, 54]. However, for a slight cost, hormone analysis may be a useful technique, particularly in completely monomorphic species or with juvenile animals as demonstrated in *Geocrina alba* [13].

Future studies on this topic may consider trialing these (and other) sex identification techniques in wild populations of DGFs, in order to better understand the expected patterns and ranges for the various measures presented here (body size, secondary sex characteristics, ultrasound profiles, and urinary hormone concentrations). Captive populations of amphibians may not demonstrate the same characteristics and patterns as their wild counterparts due to inherent differences in the captive environment, despite attempts to provide captive-reared animals with natural environmental stimuli (light cycles, natural substrate, etc.).

Due to the limited number of DGFs, both in captivity and in the wild, the animals used in this sex identification study were also part of ongoing hormone treatment trials. Although efforts were made to collect measures for this study prior to exogenous hormone treatments, the long term effect of these treatments on the measures presented here cannot be fully known. However, this situation is likely to be typical for other research groups working with endangered amphibian colonies, as they may be attempting to identify the sex of the individuals in the captive population while also attempting to start breeding efforts for extremely limited numbers of individuals. Ultimately, the use of a captive research colony of DGFs was advantageous for this study in many ways, as we were able to confirm the biological sex of the animals included in the study and could take repeated samples from individuals to assess patterns over time, which may not have been possible when working with wild populations. Determining the most effective sex identification techniques will continue to be important as the number of captive breeding populations of amphibians increases. Methods presented in this paper are highly relevant to research groups attempting to house and breed amphibians as part of *ex situ* conservation programs.

## Conclusions

A number of non-invasive and minimally invasive techniques for sex identification were trialed and compared for the weakly dimorphic captive DGF. Ultimately, each technique had specific advantages and disadvantages. Research groups must find a balance between the need for an accurate answer and the amount of time and cost they are willing to invest into sex identification, especially for monomorphic species. For adult DGFs in captivity, assessing the presence/absence of nuptial pads may provide a quick and easy answer to determine males, however the coloration of the frogs’ digits and variability in nuptial pads throughout the year may result in some inaccurate predictions. Ultrasound may be useful in identifying females with well-developed follicles when the equipment is available; however, the technique can be somewhat subjective and follicles can be difficult to discern in some cases. Analysis of urinary hormone profiles provided an accurate answer for most DGFs, but requires greater time and cost in order to achieve a result. Size measurements had limited success in the DGF and are not recommended for use. Of the methods reviewed in this paper, there is not a single 100 % accurate method for sex identification in the DGF, although overall accuracy for both nuptial pads and urinary hormone analysis (when a testosterone to estrone ratio was used) was greater than 95 %. Using multiple methods, such as assessing the presence of nuptial pads paired with hormone analysis, will likely provide the greatest accuracy.

The techniques in this study were applied and discussed specifically in regards to adult DGFs. Discerning the accuracy of these methods for sex identification in this species may improve genetic management, sustainability, and overall efficiency of captive breeding efforts for the critically endangered DGF. These techniques may also have applications to other weakly dimorphic and monomorphic anuran species and should be explored as potential tools for sex identification in other anurans.

## Abbreviations

ART, assisted reproductive technologies; BW, body weight; EIA, enzyme immunoassay; DGF, dusky gopher frog; IVF, *in vitro* fertilization; MSU, Mississippi State University; SE, standard error; SUL, snout-urostyle length; SVL, snout-vent length; T/E, testosterone to estrone ratio (concentration of urinary testosterone (ng/ml) divided by concentration of urinary estrone (ng/ml)).
